# TELEVISION VIEWING AND HEALTH IN LATE LIFE

**DOI:** 10.1093/geroni/igz038.2713

**Published:** 2019-11-08

**Authors:** Karen Fingerman, Yee To L Ng, Meng L Huo

**Affiliations:** The University of Texas at Austin, Austin, Texas, United States

## Abstract

Television viewing is a risk factor for poor physical and cognitive health. Yet, we know little about associations between current health and frequency of television viewing throughout the day. This study examined associations between multiple assessments of physical and cognitive well-being and television viewing. Participants (N = 313) from the Daily Experiences and Well-being Study completed an initial interview assessing their health and well-being. They also wore an Electronically Activated Recorders (EAR) to capture sound in the environment and an Actical to measure physical activity for 5 days. Coders rated the audiofiles for television viewing. Multilevel models revealed that participants who spent a greater proportion of time tuned into television had: higher BMI, poorer health, expended less energy, were more sedentary, and reported drinking more alcohol and eating more junk food. We discuss findings with regard to potential reciprocal influences between television viewing and poor health.

